# Predictive Validity of the Patient Health Questionnaire‐9 for Depression in Poststroke Patients: A Systematic Review and Meta‐Analysis

**DOI:** 10.1002/brb3.70464

**Published:** 2025-04-02

**Authors:** Junya Chen, Mei Chan Chong, Hmwe Nant Thin Thin, Fen Xu, Xiao Dong, Xiaoxian Yang, Ruan Jia Yin, Huimin Hong

**Affiliations:** ^1^ Department of Nursing Science Faculty of Medicine University of Malaya Kuala Lumpur Malaysia; ^2^ Department of Nursing Science Faculty of Medicine Jinhua University of Vocational Technology Jinhua Zhejiang China; ^3^ Dalian Medical University Affiliated Second Hospital Dalian China; ^4^ School of Nursing Hong Kong Polytechnic University Hong Kong China

**Keywords:** meta‐analysis, PHQ‐9, poststroke depression, sensitivity and specificity, systematic review

## Abstract

**Background:**

The Patient Health Questionnaire‐9 (PHQ‐9), known for its brevity and ease of use, is employed by researchers and clinical practitioners for poststroke depression (PSD) screening. However, the effectiveness of the PHQ‐9 in PSD screening remains to be further validated.

**Methods:**

Electronic searches were conducted in EMBASE, PubMed, Web of Science, CNKI, and Wanfang databases using keywords including stroke, depression, and PHQ‐9. The assessment tool Quality Assessment of Diagnostic Accuracy Studies‐2 was utilized to evaluate the risk of bias in diagnostic studies.

**Results:**

A total of 2049 articles were retrieved, with 9 meeting the inclusion criteria. The PHQ‐9 demonstrated pooled sensitivity and specificity of 0.84 and 0.90, respectively, and a summary receiver operating characteristic (sROC) curve of 0.93. At the 10‐cut‐off value, pooled sensitivity, specificity, and sROC were 0.77, 0.85, and 0.86, respectively. At the 9‐cut‐off value, the sensitivity, specificity, and sROC were 0.87, 0.85, and 0.92, respectively. At the 5‐cut‐off value, sensitivity, specificity, and sROC are 0.90, 0.91, and 0.96, respectively. No publication bias was identified.

**Conclusion:**

The PHQ‐9 is an effective tool for screening poststroke depressive symptoms with significant clinical utility. However, further research is needed to establish optimal diagnostic thresholds.

## Graphical abstract

This meta‐analysis confirms the high accuracy of the Patient Health Questionnaire‐9 (PHQ‐9) in screening poststroke depression. The findings highlight the impact of varying cut‐off values on sensitivity and specificity, emphasizing the need for tailored diagnostic thresholds to enhance clinical applicability across different populations.

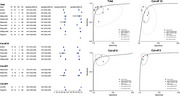

## 1 Introduction

Depression has witnessed an increasing prevalence, and poststroke depression (PSD) has been identified as a distinct subtype that occurs at each stage following a stroke (Villa et al. 2018). It constituted a common complication of strokes, particularly in recent years, witnessing a sharp rise in the overall number of PSD patients due to the expanding population of stroke survivors (Castilla‐Guerra et al. 2020). A meta‐analysis revealed that the incidence rate of PSD ranged from 11% to 41% (Guo et al. 2022). PSD was associated with a poorer functional prognosis and higher incidence of stroke recurrence and mortality rates. After a stroke, many patients experience severe and persistent functional impairments throughout their remaining lives. Typically affecting middle‐aged and older individuals, these patients frequently presented with language difficulties, limb paralysis, and other functional hindrances, which could be intimidating and disheartening for both them and their caregivers (Gorelick 2019). An association was observed between PSD and a diminished functional prognosis, as well as elevated incidence and mortality rates (Cai et al. 2019; Hu et al. 2018). Profoundly negative emotions, such as sadness, loss of interest, and despair, are prevalent across all types of depressive disorders (Sun et al. 2023). However, due to the early emphasis of stroke survivors on their differences from the past and their recovery situation, it becomes challenging to perceive changes in their emotional states (Li et al. 2020). Furthermore, symptoms like fatigue, changes in appetite, and sleep alterations are typical physical manifestations in poststroke, potentially leading to misinterpretation as false negatives (Schöttke et al. 2020). However, in current clinical practices for stroke treatment, there is a tendency to prioritize the physical recovery of patients, potentially overlooking their psychological needs.

We can use various tools for screening PSD, with representative instruments including the Beck Depression Inventory‐II (BDI‐II), the Center for Epidemiologic Studies Depression Scale (CES‐D), the Hospital Anxiety and Depression Scale (HADS), and the Montgomery Depression Rating Scale (MDRS). These tools are all self‐assessment instruments designed for adults (Chen et al. 2020; Li et al. 2017).

The Patient Health Questionnaire‐9 (PHQ‐9) is recognized as a valuable and widely used tool for assessing PSD. This self‐report instrument, consisting of nine items, provides a reliable method for evaluating the severity of depressive symptoms in stroke patients, offering advantages over other scales due to its brevity and ease of use (Strong et al. 2021). The cut‐off value of PHQ‐9 is of great significance in improving diagnostic accuracy, facilitating early intervention, enabling research comparison, achieving personalized treatment, and optimizing resource allocation. The optimal cutoff for the diagnosis of PSD has not been clearly established for different populations and different cultural backgrounds at different stages, although various studies have proposed a cutoff for PHQ‐9 in poststroke populations, usually between six and eight points, but this still needs further research. (Dajpratham et al. 2020; Turner et al. 2012). While prior studies have proposed PHQ‐9 cutoffs ranging from 6 to 13 for PSD (Okeafor and Okeafor 2023; Prisnie et al. 2016; Turner et al. 2012), these recommendations are often based on single populations, small samples, or varied methodologies. This heterogeneity limits clinical generalizability and underscores the need for a systematic synthesis of evidence to identify robust thresholds. This study aims to evaluate the screening effectiveness of PHQ‐9, focusing on two primary aspects. First, the predictive capability of PHQ‐9 is assessed by examining cut‐off values of 10, 9, and 5 points. Second, the screening performance of PHQ‐9 at these different cut‐off values is meticulously analyzed.

## 2 Method

This review is conducted according to the Cochrane Handbook for Diagnostic Test Accuracy Reviews and the 2020 PRISMA guidelines (Macaskill et al. 2010; Page et al. 2021). The protocol for this systematic review was prospectively registered with PROSPERO (ID: CRD42024524384).

### 2.1 Search Strategy and Literature Sources

In May 2024, eligible articles were systematically retrieved from five electronic databases: Web of Science, PubMed, EBSCO, CNKI, and Wanfang Database. The key search terms were depression, stroke‐related terms, and PHQ‐9. The searches for depression and poststroke conditions were based on MeSH terms (free text and MeSH, exploded). For PHQ‐9, both its full name and abbreviation were used. The search scope was expanded through free text searching, which included searches of titles, abstracts, and full texts.

### 2.2 Eligibility Criteria

Inclusion criteria of the studies were: (i) type of study, original studies reporting diagnostic accuracy (such as sensitivity and specificity) (e.g., observational studies, such as cohort or cross‐sectional studies); (ii) Type of participant, study of poststroke patients; (iii) index testing, using PHQ‐9 project research; (iv) comparison, studies comparing all types of depression screening tools with PHQ‐9 (in the meta‐analysis, depression screening tools reported in more than three studies were selected); Gold standard, studies conducted directly by trained psychiatric professionals using the diagnostic criteria for major depression as the gold standard (e.g., the fourth Diagnostic and Statistical Manual of Mental Disorders (DSM‐IV), the International Classification of Diseases (ICD‐10), the Chinese Classification of Mental Disorders (CCMD‐3), and CES‐D or structured interviews (e.g., Structured Clinical Interviews [SCID] in the DSM‐IV); (v) Studies of outcome types, including true positive (TP), false positive (FP), false negative (FN), and true negative (TN) data. From these data, sensitivity, specificity, positive and negative likelihood ratios (LRs), diagnostic odds ratios, and sROC curves were derived as outcome measures.

Exclusion criteria were: (i) retrospective studies (e.g., case‐control studies where exposure and outcome data are collected after the outcome has occurred); (ii) non‐original articles (e.g., systematic reviews, editorials, commentaries, or letters without primary data); (iii) studies using environmental monitoring systems to assess risks such as anxiety or suicide; (iv) studies that present only sensitivity or specificity and do not provide sufficient data to create a two‐by‐two contingency table; (v) studies involving subjects with comorbid psychiatric disorders (e.g., schizophrenia, bipolar disorder) or neurodegenerative diseases (e.g., Alzheimer's disease).

### 2.3 Full‐Text Screening and Data Extraction

After duplicate articles were removed, two authors (Junya Chen and Fen Xu) independently selected titles and abstracts for study screening and data extraction to confirm their potential relevance. Any disagreements between the authors were resolved through consensus. The following information was extracted from the full texts of the selected studies: year of publication, authors, location, sample size, PHQ‐9 cut‐off score, the ratio of male to female patients, age, and results such as TP, FP, FN, TN, and the scale, which was used as a gold standard in the respective papers, were recorded.

### 2.4 Quality and Risk of Bias

The quality of the selected studies was assessed using QUADAS‐2 (Quality Assessment of Diagnostic Accuracy Studies‐2) (Whiting et al. 2011). QUADAS‐2 evaluates the risk of bias and applicability through four domains: patient selection, index test, reference standard, and flow and timing. Only the applicability of the first three domains was assessed. This assessment was completed by two independent authors (Junya Chen and Fen Xu). Discrepancies between the authors were resolved through discussion to reach a consensus.

### 2.5 Statistical Analysis

Meta‐analysis was conducted using Revman 5.4.1 and Stata 18. The utilization of bivariate random‐effects models facilitated the assessment of screening accuracy and study heterogeneity. Screening accuracy was evaluated by combining sensitivity, specificity, positive and negative LRs, 95% confidence intervals (CIs), and the area under curve (AUC) the summary receiver operating characteristic (sROC). The testing precision was described through the analysis of AUC and Q* index values. AUC values were interpreted as follows: AUC = 0.5 indicated no discrimination; AUC between 0.5 and 0.7 indicated low accuracy; AUC between 0.7 and 0.9 indicated moderate accuracy; AUC between 0.9 and 1 indicated high accuracy; and AUC = 1 indicated perfect test performance (Greiner et al. 2000). The Q* index represented the point on the ROC curve where sensitivity equals specificity, with a value of 1 denoting 100% accuracy (Walter 2002). The sensitivity and specificity of PHQ‐9 were illustrated using forest plots and sROC curves. Additionally, PHQ‐9 screening performance across various thresholds was summarized to assess its diagnostic utility. Heterogeneity among included studies was quantified using Cochrane's *Q* statistic and *I* ^2^ statistic with Stata 18. If significant I^2^ is detected in the study, a subgroup analysis will be conducted for each included study (*I* ^2^ around 25% may be considered low, 50% moderate, and 75% significant) (Higgins, Thompson, et al. 2003; Higgins, Thomas, et al. 2024). Reference standard, sample size, country, and cut‐off value intervention will be considered as independent variables to identify potential sources of heterogeneity. However, since most studies do not explicitly specify or classify the stroke phase of the participants, subgroup analysis will not be performed based on this factor. Publication bias in included studies was assessed using Deek's funnel plot (Du et al. 2017). All hypothesis tests were two‐sided with statistical significance set at *p* < 0.05.

## 3 Result

**FIGURE 1:**
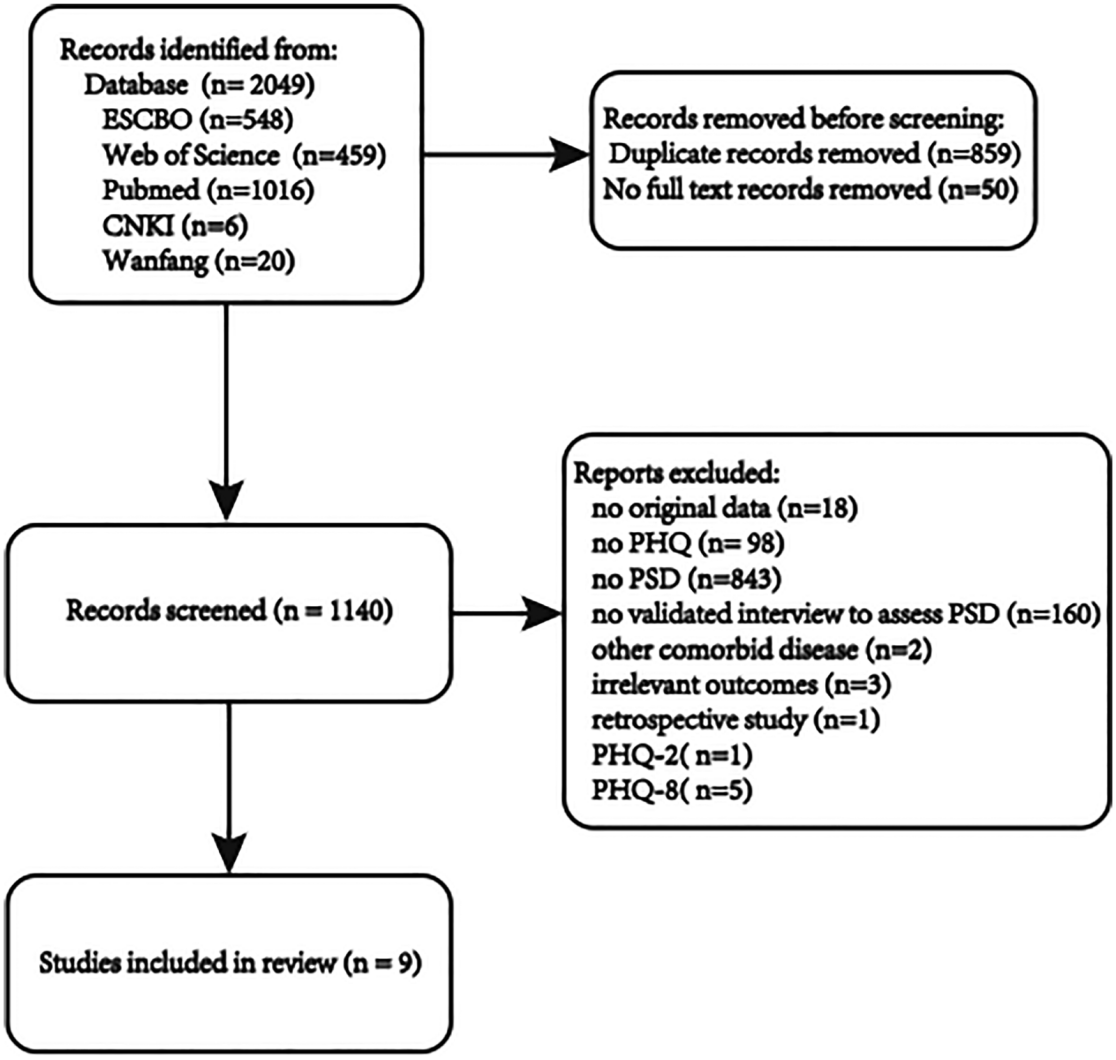
Flow diagram of article selection.

A total of 2049 articles were retrieved from electronic databases. Duplicate articles (*n* = 859) were excluded. The inclusion and exclusion criteria were applied to the titles and abstracts of 1190 articles, among which 50 were conference papers or abstracts only. Nine studies were retained for quantitative synthesis, while 1131 articles (99.2%) were excluded. The study selection process is detailed in the flowchart, as shown in Figure 1.

### 3.1 Data Extraction and Critical Appraisal

**FIGURE 2:**
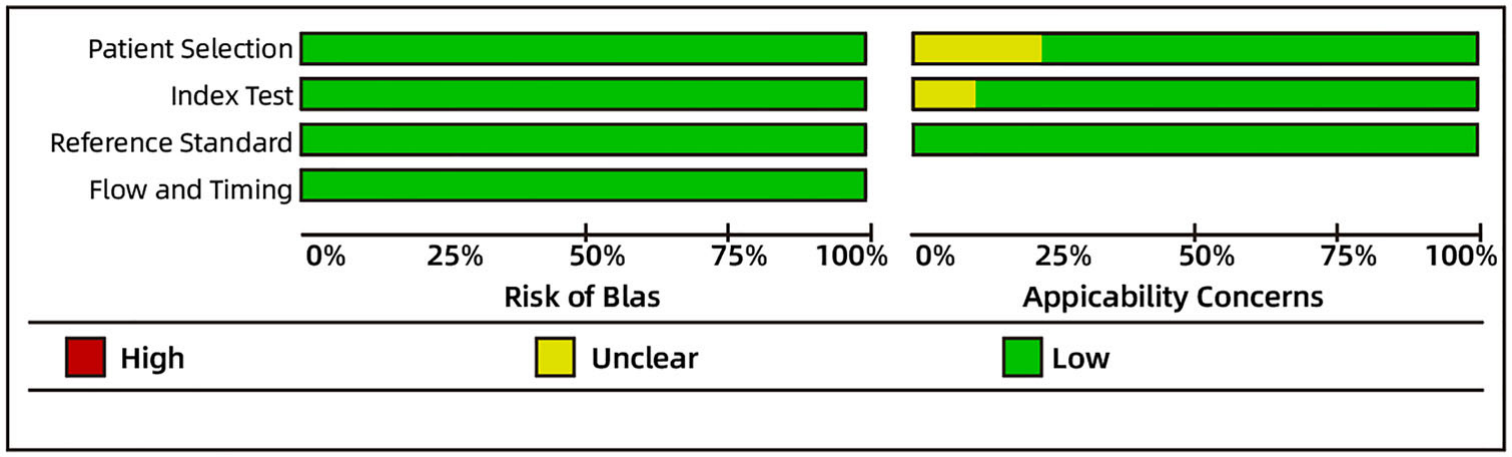
Risk‐of‐bias assessment of the included studies for shaking chills using the QUADAS‐2 tool.

All eligible published studies were independently evaluated by at least two reviewers using the QUADAS‐2 tool to assess methodological quality (Figure 2). Data was extracted independently by at least two authors, who constructed 2 × 2 tables to calculate the primary outcomes, sensitivity, and specificity. To maximize the use of available data, the most consistently reported and recommended cut‐off points for each scale were extracted.

### 3.2 Publication Bias

**FIGURE 3:**
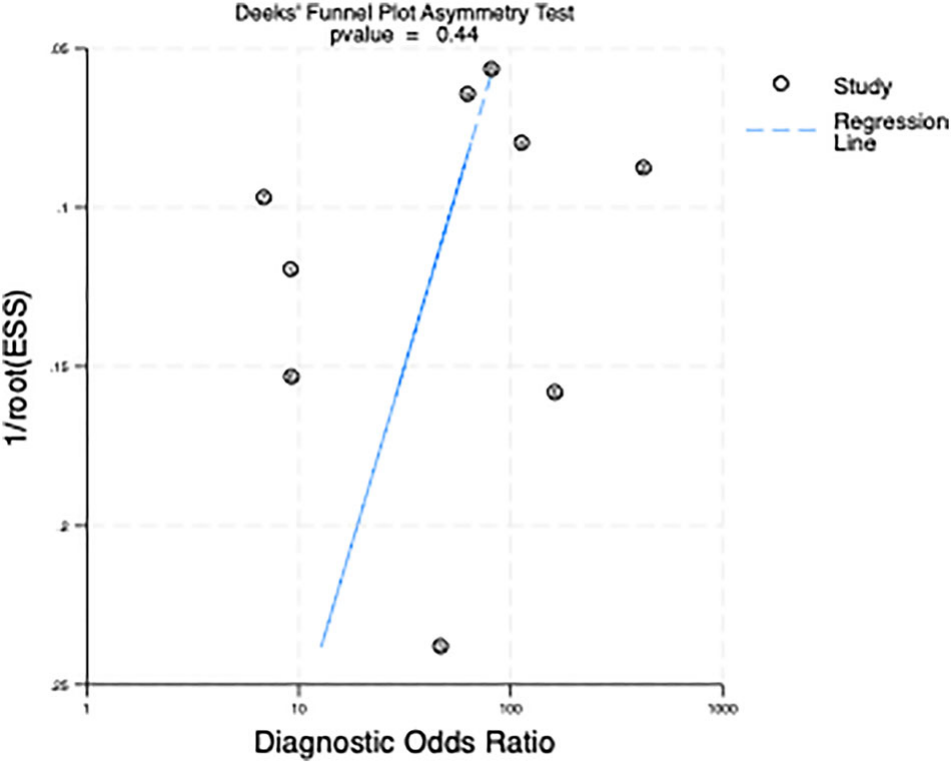
Deek funnel plot showing publication bias.

The Deek funnel plot (Figure 3) demonstrates a slope coefficient of 0.44, indicating the absence of publication bias in the included studies. While Deek's funnel plot indicated no significant publication bias, the interpretation of this result should consider the influence of small sample sizes. Studies with smaller samples are more likely to produce extreme effect sizes (e.g., higher sensitivity/specificity) due to random error, which may skew funnel plot symmetry. Additionally, the inclusion of only nine studies limits the power of the funnel plot analysis. These factors underscore the need for caution when interpreting the pooled estimates, particularly for subgroups with fewer studies.

### 3.3 Summary Included Studies

A total of 1,140 potential studies were identified. From these, nine validation studies met the inclusion criteria and provided sufficient data (de Man‐van Ginkel et al. 2012; Luo 2019; Mikami et al. 2021; Mikami et al. 2021; Okeafor and Okeafor 2023; Prisnie et al. 2016; Turner et al. 2012; K. Williams et al. 2020; L. Williams et al. 2005; Yang 2016) (Table 1). The diagnostic properties of the PHQ‐9 are examined in these studies. Two studies involve inpatient settings (Luo 2019; Mikami et al. 2021), whereas the others are conducted in outpatient clinics, primary care centers, and similar environments. Five studies specifically looked at convalescent stroke patients (> 1 month) (de Man‐van Ginkel et al. 2012; Luo 2019; Mikami et al. 2021; Prisnie et al. 2016; Turner et al. 2012; L. Williams et al. 2005; Yang 2016), and two studies did not report the duration of the patient's disease. Patient ages ranged from 20 to 91 years and were reported in six studies (de Man‐van Ginkel et al. 2012; Luo 2019; Mikami et al. 2021; Okeafor and Okeafor 2023; Turner et al. 2012; Yang 2016), and four studies reported mean ages between 60.1 and 70.6 years (de Man‐van Ginkel et al. 2012; Prisnie et al. 2016; Turner et al. 2012; Wang et al. 2018). The studies were published across eight countries, with two from China (Luo 2019; Yang 2016) and seven from other countries (de Man‐van Ginkel et al. 2012; Mikami et al. 2021; Okeafor and Okeafor 2023; Prisnie et al. 2016; Turner et al. 2012; Wang et al. 2018; L. Williams et al. 2005). Seven studies included more than 100 patients (de Man‐van Ginkel et al. 2012; Luo 2019; Okeafor and Okeafor 2023; Prisnie et al. 2016; Turner et al. 2012; Wang et al. 2018; L. Williams et al. 2005). DSM‐IV was used as the gold standard in six studies (de Man‐van Ginkel et al. 2012; Mikami et al. 2021; Okeafor and Okeafor 2023; Prisnie et al. 2016; Turner et al. 2012; L. Williams et al. 2005), CCMD‐3 in two Chinese studies (Luo 2019; Yang 2016), and CES‐D in one study (Wang et al. 2018). The proportion of depression diagnosed in the screened populations varied significantly, ranging from 9% to 79.1%. However, six studies did not specify whether the gold standard assessors were blinded to the PHQ‐9 results (Luo 2019; Mikami et al. 2021; Okeafor and Okeafor 2023; Turner et al. 2012; L. Williams et al. 2005; Yang 2016). All nine studies reported the presence or absence of PSD. The optimal cut‐off values for PHQ‐9 differed among the studies, with two studies evaluating the efficacy of different cut‐off values (Prisnie et al. 2016; Turner et al. 2012). For the meta‐analysis, the cut‐off value deemed most efficient by the authors was selected from these two studies.

**TABLE 1:** Characteristics of selected studies (*N* = 9).

											2 × 2 value				Value (95% confidence interval)		
Reference	Country	Prevalence (%)	Blind	Year	Study design	Sample	Cut‐off value (best)	Male/female	Age range(Mean)	Reference standard	*TP*	*FP*	*FN*	*TN*	Sensitivity	Specificity	Setting
de Man‐van Ginkel et al. (2012)	The Netherlands	12.2	Yes	2012	Cross‐sectional	164	≥ 10	97/67	20–79 (70.6)	DSM‐IV ICD‐10	15	35	5	107	0.75 (0.51–0.91)	0.75 (0.67–0.82)	Rehabilitation 6–8 weeks
Luo et (2019)	China	34.7	Unknown	2019	Cross‐sectional	144	≥ 5	105/39	36–84	CCMD‐3	41	1	9	93	0.82 (0.69–0.91)	0.99 (0.94–1.00)	Rehabilitation 1–6 months
Mikami et al. (2021)	Japan	79.1	Unknown	2020	Cross‐sectional	48	≥ 9	37/11	20–85	DSM‐IV (SCID)	35	1	3	4	0.92 (0.79–0.98)	0.80 (0.28–0.99)	With 6 weeks
Okeafor and Okeafor (2023)	Nigeria	26.9	Unknown	2023	Cross‐sectional	197	≥6	132/65	35–76	DSM‐IV (SCID)	47	10	6	144	0.89 (0.77–0.96)	0.94 (0.88–0.97)	Unknown
Prisnie et al. (2016)	Canada	9	Yes	2016	Cross‐sectional	121	≥ 9	53/68	Unknow (60.1)	DSM‐IV (SCID)	9	10	2	100	81.8 (48.2–97.7)	91.3 (84.1–95.9)	Rehabilitation
							≥ 10				9	8	2	102	81.8 (48.2–97.7)	93.2 (86.5–97.2)	
							≥ 11				9	5	2	105	81.8 (48.2–97.7)	96.1 (90.4–98.9)	
							≥ 12				9	4	2	106	81.8 (48.2–97.7)	97.1 (97.1–99.4)	
							≥ 13				9	4	2	106	81.8 (48.2–97.7)	97.1 (97.1–99.4)	
							≥ 14				8	4	3	106	72.7 (39.0–94.0)	97.1 (97.1–99.4)	
Turner et al. (2012)	Australia	18.1	Unknown	2012	Cross‐sectional	72	≥ 7	38/34	25–91 (66.7)	DSM‐IV	11	22	2	37	0.85 (0.55–0.98)	0.63 (0.49–0.75)	>3 weeks
							≥ 9				10	15	3	44	0.77 (0.46–0.95)	0.75 (0.62–0.85)	
							≥ 10				9	13	4	46	0.69 (0.39–0.91)	0.78 (0.65–0.88)	
Wang et al. (2018)	America	23.8	Yes	2018	Cross‐sectional	147	≥ 10	123/24	Unknown (69.6)	CES‐D	18	15	17	97	0.51 (0.34–0.69)	0.87 (0.79–0.92)	Unknown
L. Williams et al. (2005)	India	45.8	Unknown	2005	Cross‐sectional	316	≥ 10	171/145	Unknown	DSM‐IV (SCID)	132	19	13	152	0.91 (0.85–0.95)	0.89 (0.83–0.93)	1–2 months
Yang (2016)	China	36.9	Unknown	2016	Cross‐sectional	260	≥ 5	144/116	46–89	CCMD‐3	85	18	11	146	0.89 (0.80–0.94)	0.89 (0.83–0.93)	Rehabilitation 1–6 months

### 3.4 Meta‐Analysis of the Accuracy of the PHQ‐9 in Diagnosing PSD

#### 3.4.1 Heterogeneity Analysis and Subgroup Analysis

**FIGURE 4:**
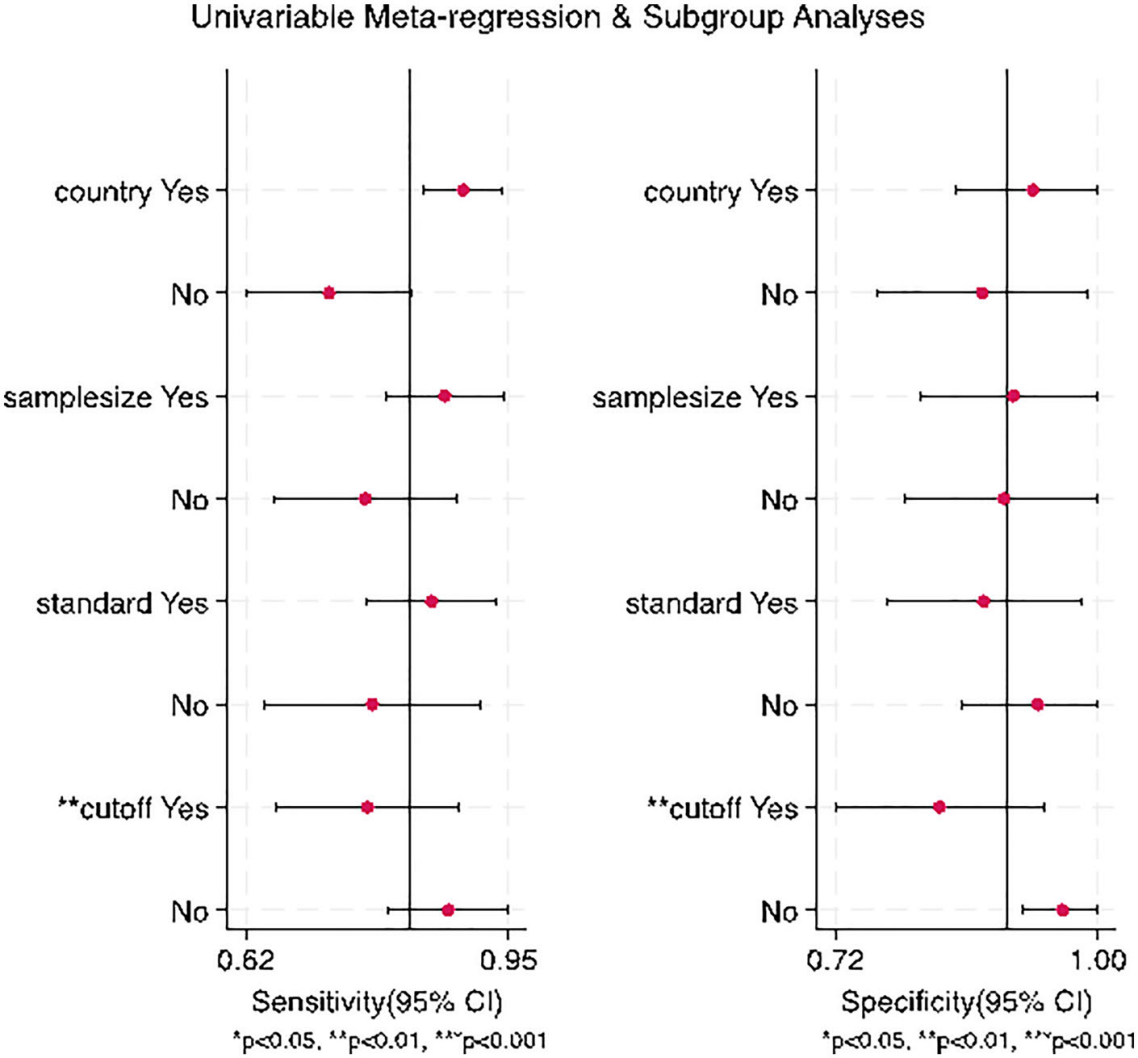
Subgroup analysis and meta‐regression of selected studies.

The results indicate high heterogeneity (*Q* = 15.338, *I* ^2^ = 88.7%), necessitating the use of a random‐effects model to combine the effect sizes. Due to the high heterogeneity, a further meta‐regression analysis was conducted. The results indicated that sample size, reference standards, and country had no statistically significant effect on heterogeneity, whereas cut‐off value had a significant impact (*p* < 0.01) (Figure 4).

#### 3.4.2 Combined Effect Analysis

**FIGURE 5:**
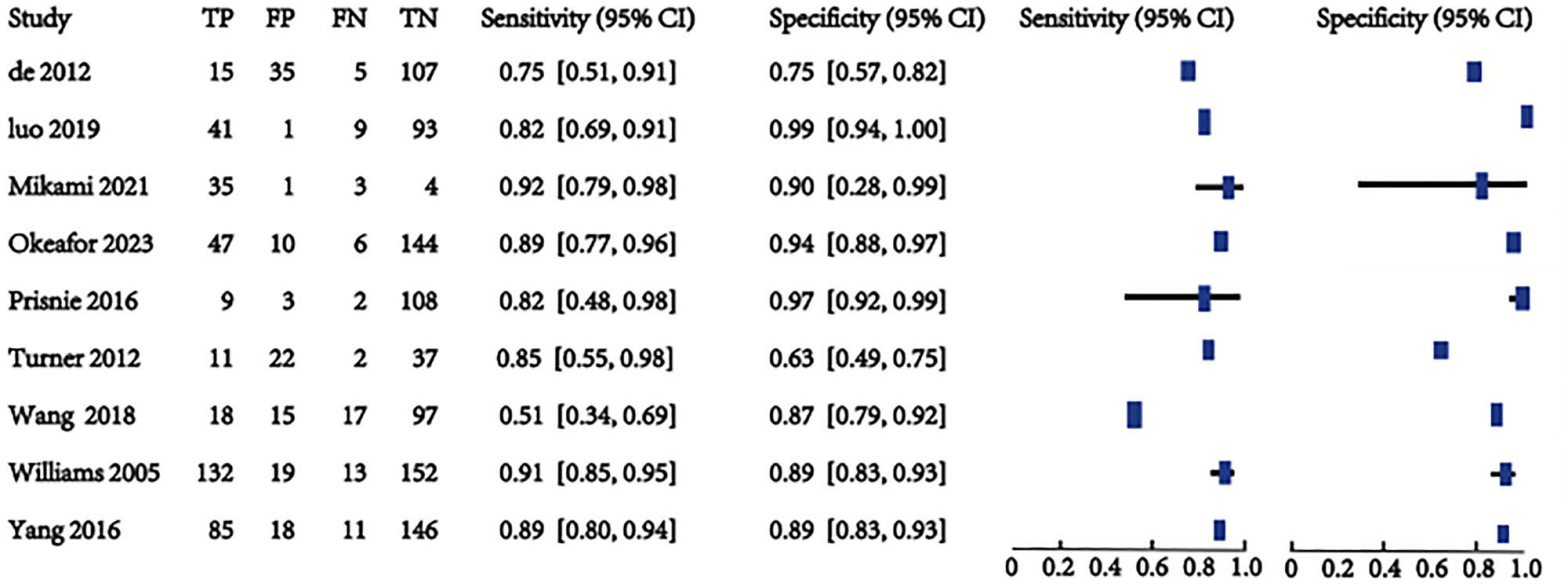
Sensitivity and specificity in total subjects.

**FIGURE 6:**
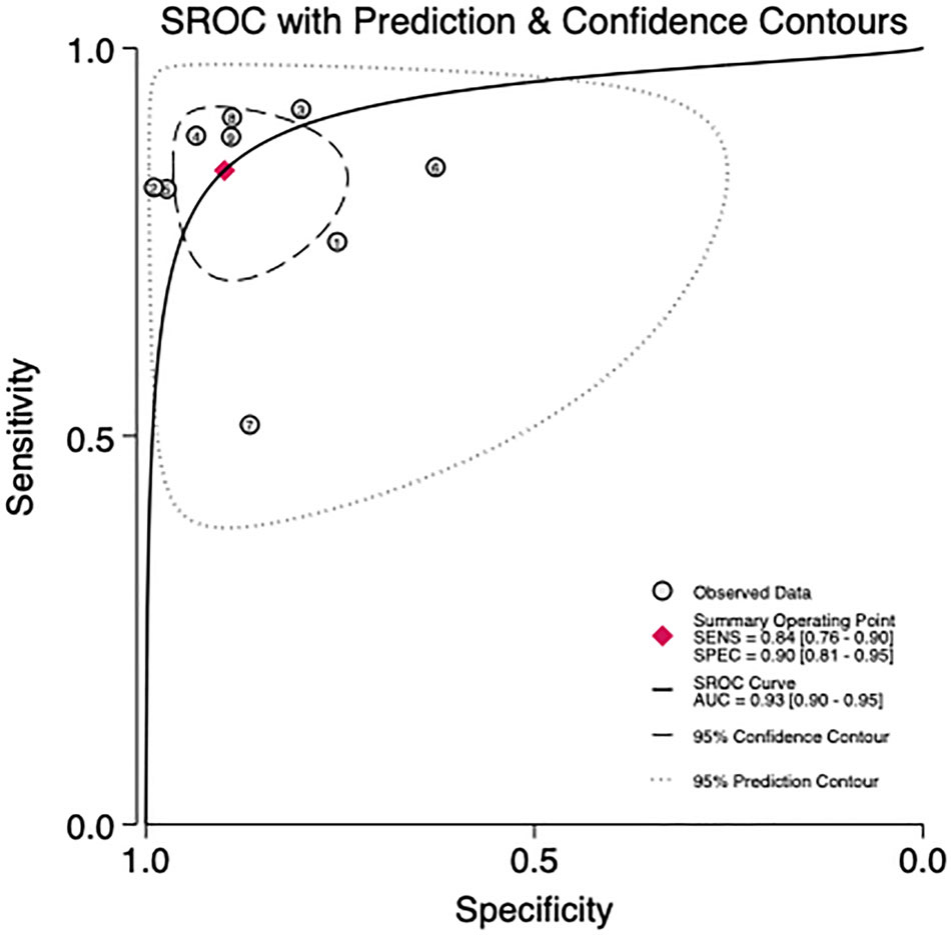
sROC curve in total subjects.

Nine studies involving 1469 patients are being analyzed to assess the predictive validity of the PHQ‐9 (Figures 5 and 6). The prevalence of PSD is 31.4%. Sensitivity ranges from 0.51 to 0.92, and specificity ranges from 0.63 to 0.99. The combined sensitivity and specificity from the meta‐analysis are 0.84 (95% CI 0.76–0.90) and 0.90 (95% CI 0.81–0.95), respectively. The sROC AUC is 0.93 (SE = 0.01), and the Q* value is 0.73 (SE = 0.02).

**FIGURE 7:**
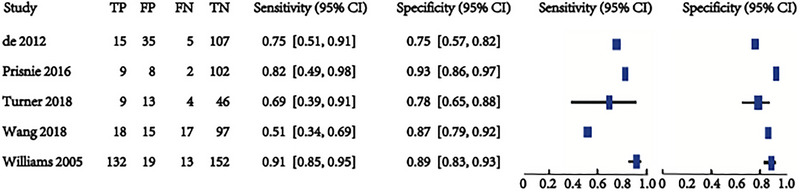
Sensitivity and specificity in subjects at the cut‐off of 10.

**FIGURE 8:**
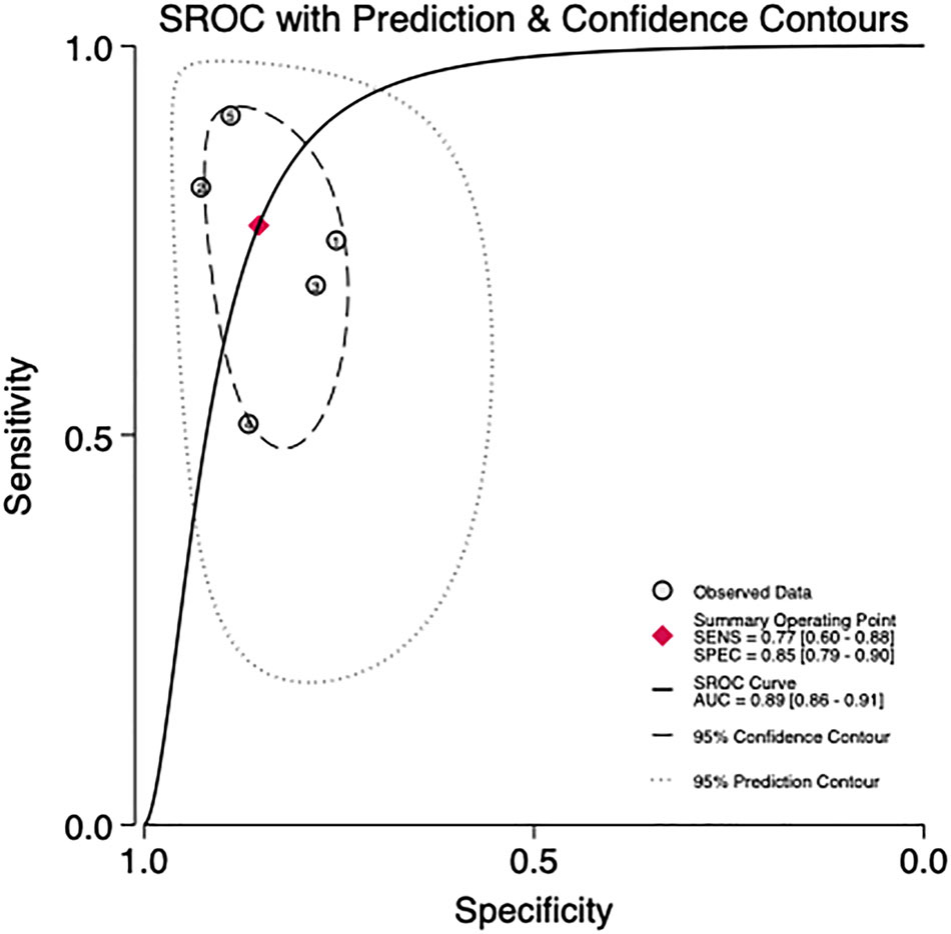
sROC curve in subjects at the cut‐off of 10.

Five studies involving 820 patients are being analyzed to assess the predictive validity of the PHQ‐9 at a cut‐off value of 10 (Figures 7 and 8). The prevalence of PSD is 27.3%. Sensitivity ranges from 0.51 to 0.91, and specificity ranges from 0.75 to 0.93. The combined sensitivity and specificity from the meta‐analysis are 0.77 (95% CI 0.60–0.88) and 0.85 (95% CI 0.79–0.90), respectively. The sROC AUC is 0.86 (SE = 0.02), and the Q* value is 0.62 (SE = 0.03).

**FIGURE 9:**
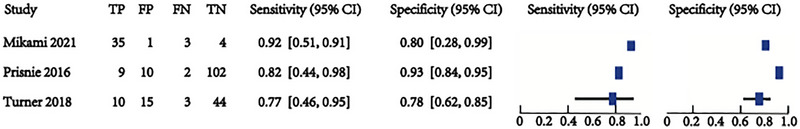
Sensitivity and specificity in subjects at the cut‐off of 9.

**FIGURE 10:**
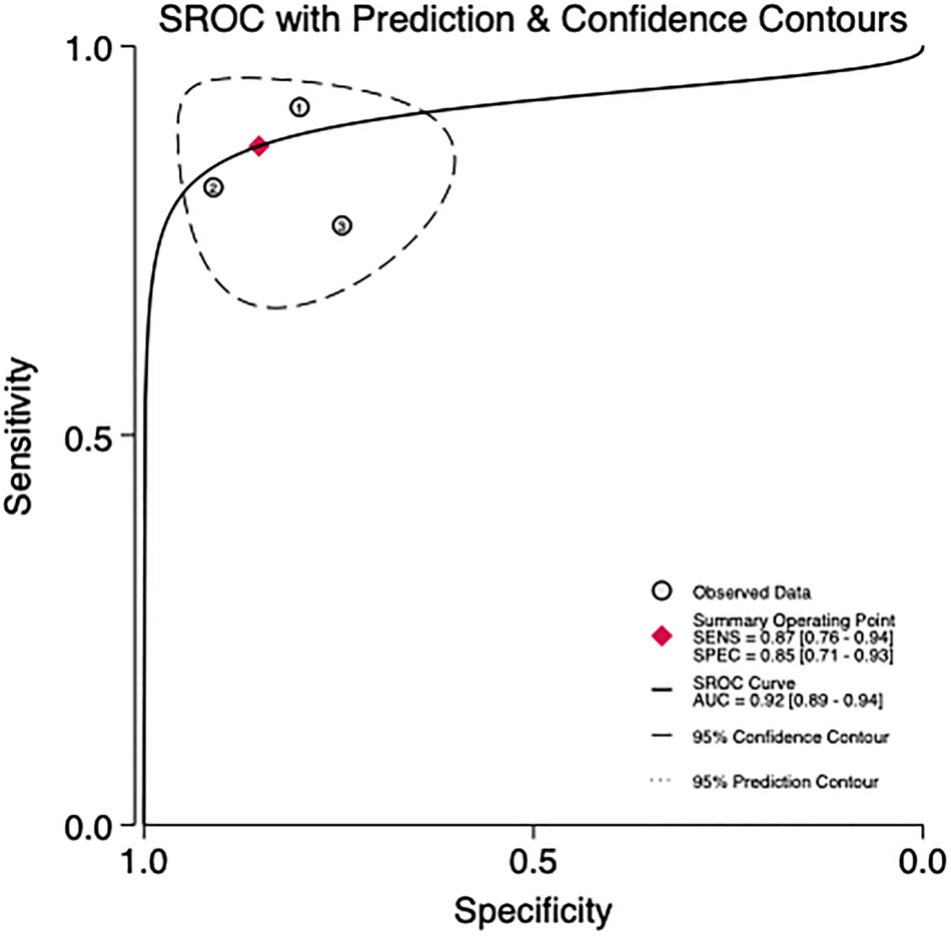
sROC curve in subjects at the cut‐off of 9.

Three studies involving 241 patients are being analyzed to assess the predictive validity of the PHQ‐9 at a cut‐off value of 9 (Figures 9 and 10). The prevalence of PSD is 25.7%. Sensitivity ranges from 0.82 to 0.92, and specificity ranges from 0.78 to 0.93. The combined sensitivity and specificity from the meta‐analysis are 0.87 (95% CI 0.76–0.94) and 0.85 (95% CI 0.89–0.94), respectively. The sROC AUC is 0.92 (SE = 0.03), and the Q* value is 0.72 (SE = 0.05).

**FIGURE 11:**
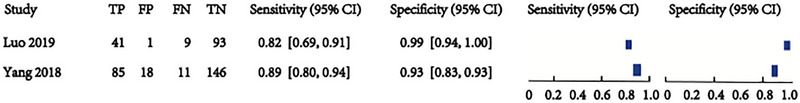
Sensitivity and specificity in subjects at the cut‐off of 5.

**FIGURE 12:**
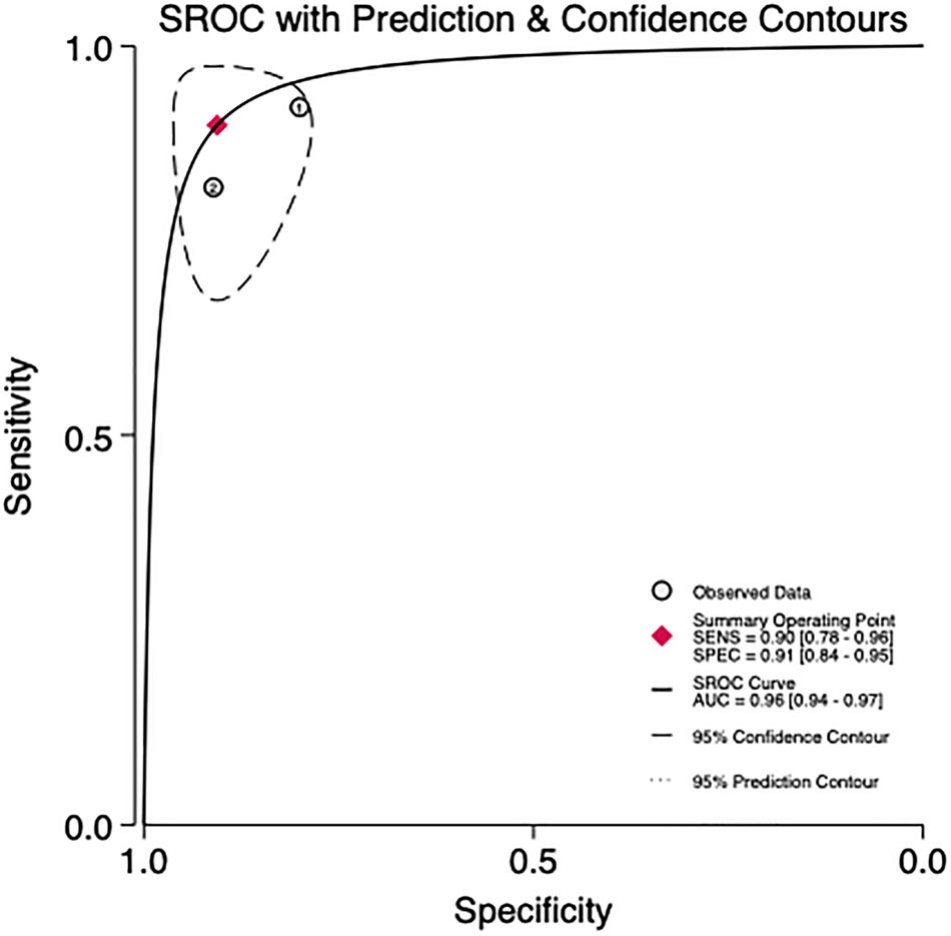
sROC curve in subjects at the cut‐off of 5.

Two studies involving 404 patients are being analyzed to assess the predictive validity of the PHQ‐9 at a cut‐off value of 5 (Figures 11 and 12). The prevalence of PSD is 36.1%. Sensitivity ranges from 0.82 to 0.89, and specificity ranges from 0.93 to 0.99. The combined sensitivity and specificity from the meta‐analysis are 0.90 (95% CI 0.78–0.96) and 0.91 (95% CI 0.84–0.95), respectively. The sROC AUC is 0.96 (SE = 0.01), and the Q* value is 0.81 (SE = 0.03).

**FIGURE 13:**
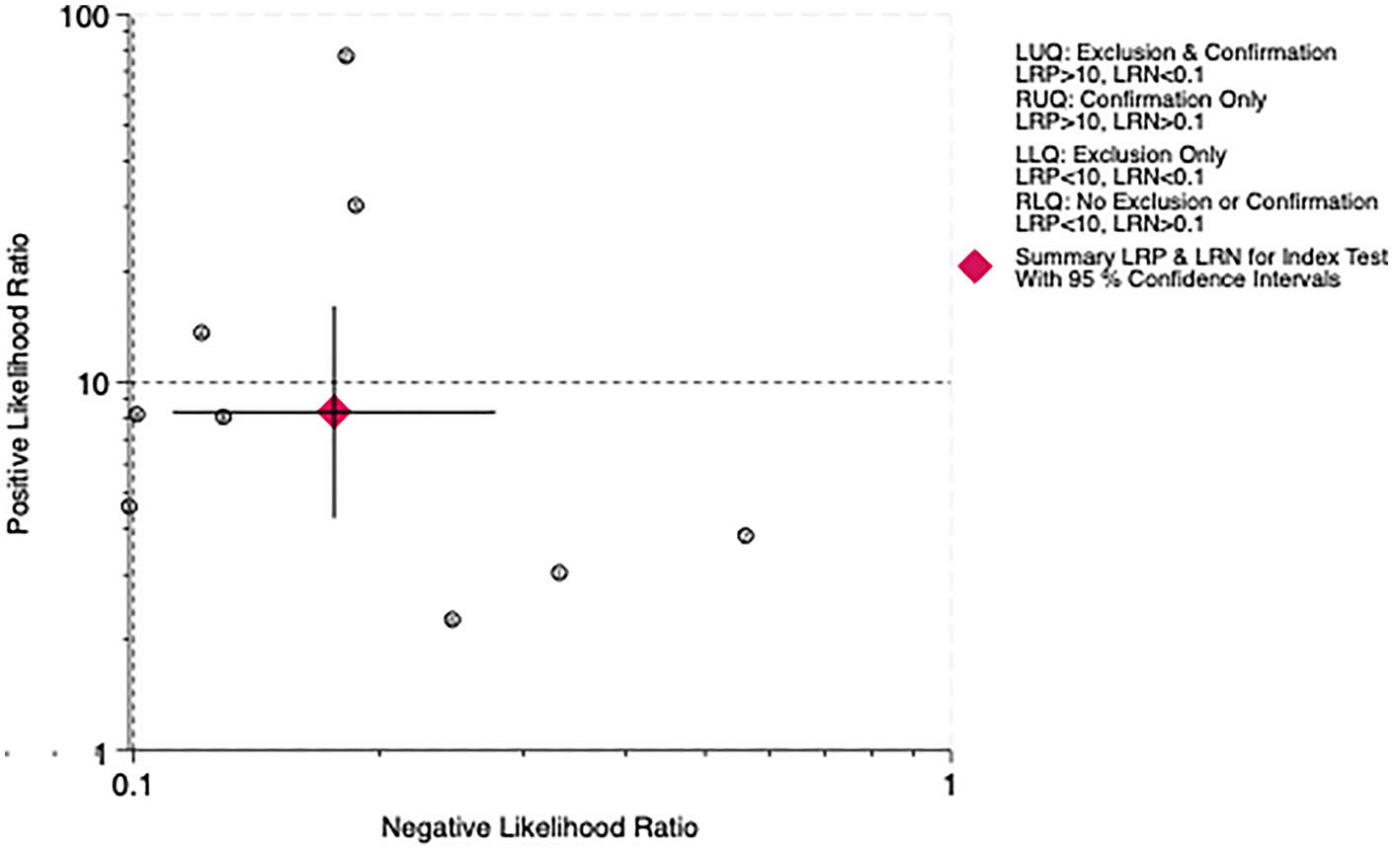
Distribution scatter diagram of the likelihood ratio (LR+/LR−) of all subjects.

**FIGURE 14:**
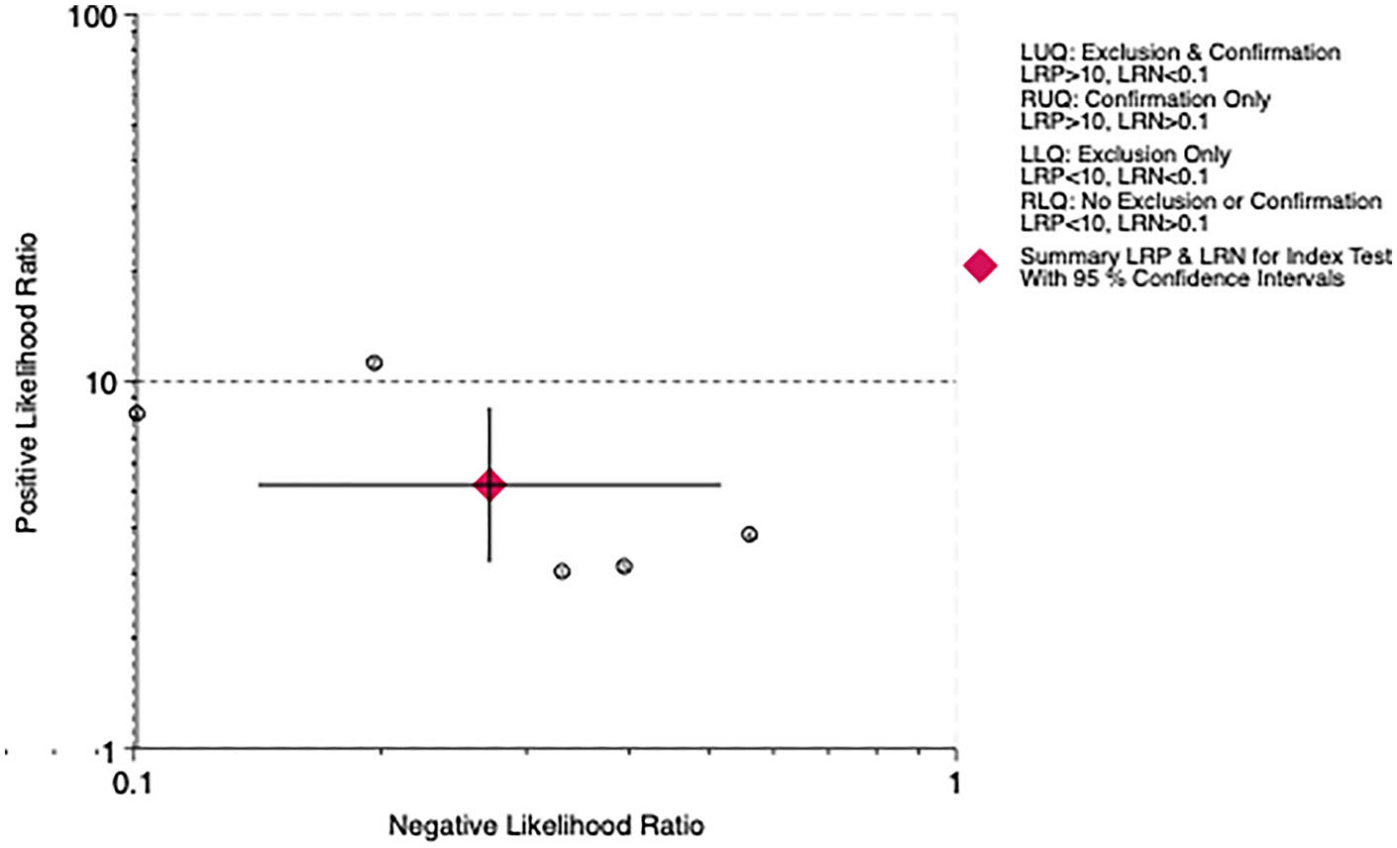
Distribution scatter diagram of the likelihood ratio (LR+/LR−) in subjects at the cut‐off of 10.

The overall LR scatter plot and the LR scatter plot at the 10‐point cutoff value show that the summary estimate of the 95% confidence interval is in the lower right quadrant, indicating that the PHQ‐9 has poor overall accuracy in diagnosing PSD (Figures 13 and 14).

#### 3.4.3 Fagan Nomogram Analysis

**FIGURE 15:**
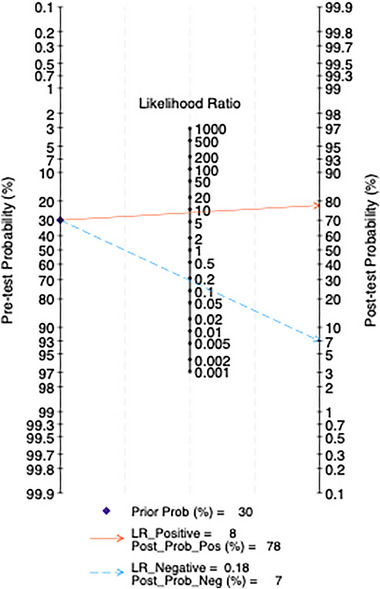
Fagan nomogram in total subjects.

**FIGURE 16:**
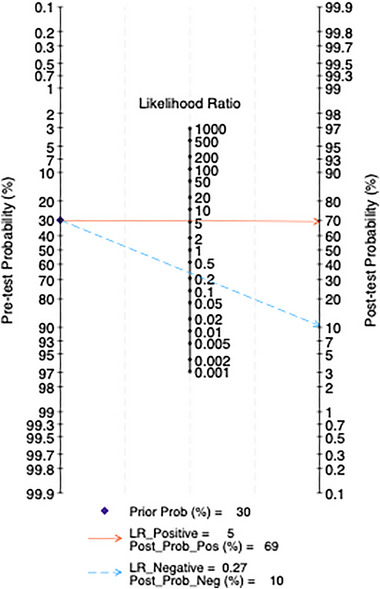
Fagan nomogram in subjects at the cut‐off of 10.

All studies are included, and clinical scenarios are simulated based on a predicted probability corresponding to an average incidence rate of 30%. This simulation results in a posterior probability of 78% for positive test outcomes and a negative LR of 0.16, with a posterior probability of 7% for negative outcomes. In the five studies using a cutoff score of 10, clinical scenarios are similarly simulated based on a predicted probability of an average incidence rate of 30%. This leads to a posterior probability of 69% for positive test outcomes and a negative LR of 0.27, with a posterior probability of 7% for negative outcomes (Figures 15 and 16).

## 4 Discussion

After a stroke, PSD is a common psychological issue, with reported incidence rates of around one‐third across different phases poststroke (Towfighi et al. 2017). PHQ‐9 is widely utilized as a depression assessment tool in poststroke research, often used to determine both the presence and severity of PSD (Dajpratham et al. 2020). Due to patient noncompliance during acute phases, research predominantly focuses on the subacute phase (Kouwenhoven et al. 2011). Given the importance of early detection and intervention for PSD, studies typically span from 1 month to 1 year poststroke, involving predominantly first‐time stroke patients, a detail confirmed across nine included studies. The use of PHQ‐9 in poststroke populations was first documented in 2005 (L. Williams et al. 2005). However, consensus on its optimal cutoff values for diagnosing PSD and population‐specific differences remains uncertain. Despite this ambiguity, PHQ‐9's brevity and convenience have led many studies to adopt it as a gold standard (Yue et al. 2022). To enhance the precision of PSD diagnosis using PHQ‐9, this study undertakes a systematic review and meta‐analysis using standardized methods. It summarizes and analyzes the diagnostic performance of PHQ‐9, particularly exploring its accuracy at different cutoff values, aiming to optimize its utility in PSD assessment in research settings. Firstly, the selected studies were evaluated for quality using QUADAS‐2. PHQ‐9, as a self‐assessment tool with a quantifiable scoring system, minimizes procedural bias during testing (Haq et al. 2010; Park and Kim 2023). Therefore, adherence to blinded criteria is crucial in assessing literature quality. Given that this study includes only nonstructured or semi‐structured interviews with psychiatric professionals, there is no deviation in the reference standard for correctly classifying severe depression. Three studies reported blinding methods, allowing for a comprehensive evaluation of other indicators with a relatively low risk of bias. Given the high heterogeneity among the selected studies, a subgroup analysis was conducted, identifying variations in cutoff values as the primary source of heterogeneity. Consequently, studies with different cutoff values were specifically analyzed.

The PHQ‐9 demonstrates high accuracy in the overall meta‐analysis, with an sROC AUC of 0.93 (SE = 0.01). Our meta‐analysis addresses a critical gap by aggregating data across diverse populations and settings. For instance, while earlier single‐center studies proposed higher cutoffs (e.g., 10–13) to maximize specificity (Prisnie et al. 2016; L. Williams et al. 2005), our pooled analysis demonstrates that a lower cutoff of five achieves superior AUC (0.96) in Chinese cohorts. This contrasts with Turner et al. 2012, who advocated for a cutoff of 8 in Western populations, highlighting the influence of cultural and demographic factors on optimal thresholds. The highest accuracy is observed at a cutoff score of 5, achieving an sROC AUC of 0.96 (SE = 0.01). However, this finding is based on only two studies, both conducted on Chinese populations, which is insufficient to draw definitive conclusions. While the PHQ‐9 demonstrated high pooled sensitivity (0.84) and specificity (0.90), the limited sample sizes in subgroup analyses (e.g., two studies at a cutoff of 5) may reduce the reliability of these estimates. Smaller samples increase the risk of overfitting and reduce statistical power, potentially inflating performance metrics. For instance, the AUC of 0.96 at the cutoff of 5, derived from only two studies, may not generalize to broader populations. Future studies with larger, diverse cohorts are needed to validate these findings and ensure stability in sensitivity and specificity estimates. At cut‐off scores of 10 and 9, the accuracy is AUC 0.92 (SE = 0.02) and AUC 0.89 (SE = 0.03), respectively. To our knowledge, this is the first meta‐analysis to systematically evaluate PHQ‐9's accuracy across multiple cutoffs (5, 9, 10) using a standardized diagnostic framework. Unlike previous reviews focusing on a single threshold, our stratified analysis reveals that lower cutoffs (e.g., five) may enhance sensitivity without compromising specificity in specific subgroups, a finding not previously reported. Due to the limited number of studies, further research is necessary to determine the optimal cut‐off score for the diagnostic accuracy of PHQ‐9. The observed trade‐off between sensitivity and specificity at varying PHQ‐9 cutoff values underscores critical clinical implications. A lower cutoff (e.g., 5) prioritizes sensitivity (0.90), minimizing missed cases of PSD, which is crucial in settings where early intervention is paramount. However, this comes at the cost of reduced specificity (0.91), potentially increasing FPs and overburdening healthcare systems with unnecessary referrals or treatments. Additionally, comorbid conditions such as cardiometabolic risks, which are closely linked to body composition changes (Arslan 2023), may further complicate the interpretation of depressive symptoms in poststroke patients. Conversely, a higher cutoff (e.g., 10) enhances specificity (0.85), reducing false alarms but risking underdiagnosis, particularly in populations where PSD comorbidities exacerbate functional impairments. Notably, comorbidities such as chronic kidney disease (CKD) may further complicate the interpretation of PHQ‐9 scores. For instance, in hemodialysis patients, malnutrition and systemic inflammation are key predictors of mortality (Yaprak et al. 2023), and these factors may also modulate depressive symptom reporting or severity. Future studies should explore whether PHQ‐9 performance varies in populations with complex medical profiles, such as those requiring hemodialysis, where both physiological and psychosocial stressors intersect. Clinicians must weigh these trade‐offs based on context: in resource‐limited environments or for initial screening, a lower cutoff may be justified to capture at‐risk patients, while higher cutoffs could be reserved for confirmatory assessments or research settings requiring precision. Future guidelines should consider stratified approaches, tailoring cutoff selection to clinical priorities (e.g., acute vs. rehabilitation phases) and patient characteristics (e.g., cultural differences in symptom reporting).

The LR scatter plots both indicate the low diagnostic performance of the PHQ‐9. Therefore, this study incorporates the Fagan nomogram for further analysis. The Fagan nomogram reveals that the overall posttest probability across all studies is 78%, while the posttest probability at the 10‐point cutoff is 69%. Both negative predictive values are less than 10%, suggesting that the PHQ‐9 performs well in predicting patients with PSD but tends to falsely diagnose patients without PSD as having PSD. Furthermore, the generalizability of our findings may be constrained by the demographic and geographic distribution of included studies. For example, the high accuracy observed at a cutoff of 5 was derived exclusively from Chinese populations, raising questions about cross‐cultural validity. Cultural differences in symptom reporting, stigma around mental health, and varying healthcare contexts could influence PHQ‐9 performance. Further research in diverse ethnic, linguistic, and clinical settings is critical to establish universally optimal cutoffs and ensure equitable applicability in global stroke care.

## 5 Limitation

This study has several limitations. Firstly, the cut‐off value of PHQ‐9 is determined based on the optimal values proposed by various studies. While most studies recommend a cut‐off value of 10 for PHQ‐9, Pristine's study identifies 13 as the optimal threshold (Prisnie et al. 2016). However, some studies have used unique cut‐off values such as 6 (Okeafor and Okeafor 2023). Secondly, subgroup analysis is limited by the insufficient number of studies using cut‐off values of 9 and 5, making it difficult to determine their predictive performance and inhibitory effects at these thresholds. Thirdly, this meta‐analysis does not conduct subgroup analyses on data from the rehabilitation and sequelae phases. Estimating the sensitivity and specificity of PHQ‐9 for PSD across different cut‐offs requires including patients at various stages of the disease. Lastly, firsthand data were not obtained in this study, and the analysis of the diagnostic efficacy of various cut‐off values requires the collection of such data. This will be the focus of our future research.

## 6 Conclusion

This study, based on nine rigorously designed low‐risk bias studies, demonstrates that the PHQ‐9 serves as an effective screening tool for PSD. The sROC AUC of PHQ‐9 is 0.93, indicating high accuracy. By synthesizing global evidence, this study advances the field by demonstrating that PHQ‐9's diagnostic performance varies significantly across cutoffs and populations. Our findings challenge the “one‐size‐fits‐all” approach to PSD screening and underscore the necessity for culturally tailored thresholds, a perspective not comprehensively addressed in prior research. The PHQ‐9 is an excellent screening tool in primary care settings and communities. Based on current evidence, we propose pragmatic recommendations: (1) In primary care or community screening, where early detection is critical, a cutoff of 5–6 may optimize sensitivity, ensuring timely referral for comprehensive evaluation. (2) In specialized stroke rehabilitation units, a cutoff of 9–10 could balance sensitivity and specificity, aligning with the need for accurate diagnosis amid higher baseline PSD prevalence. (3) For research protocols prioritizing diagnostic certainty, a cutoff of 10 should be adopted, albeit with supplemental assessments to mitigate false negatives. These suggestions acknowledge the limitations of existing data and emphasize the need for context‐driven adaptation, particularly in diverse cultural and clinical populations. However, further large‐scale studies are required to determine the optimal cut‐off value for diagnosing PSD with PHQ‐9.

## Author Contributions

**Junya Chen**: writing – original draft, conceptualization, data curation, funding acquisition, supervision. **Meichan Chong**: conceptualization, writing – review and editing, supervision. **Nant Thin Thin Hmwe**: writing–review and editing. **Fen Xu**: data curation. **Xiao Dong**: formal analysis. **Jia Yin Ruan**: formal analysis. **Xiaoxian Yang**: formal analysis. **Huimin Hong**: formal analysis.

## Conflicts of Interest

The authors declare no conflicts of interest.

### Peer Review

The peer review history for this article is available at https://publons.com/publon/10.1002/brb3.70464.

## Data Availability

Data sharing is not applicable to this article as no datasets were generated or analyzed during the current study.
